# Role of DNA-detection–based tools for monitoring the soil-transmitted helminth treatment response in drug-efficacy trials

**DOI:** 10.1371/journal.pntd.0007931

**Published:** 2020-02-06

**Authors:** Javier Gandasegui, María Martínez-Valladares, Berta Grau-Pujol, Alejandro J. Krolewiecki, Rafael Balaña-Fouce, Woyneshet Gelaye, Lisette van Lieshout, Stella Kepha, Inácio Mandomando, José Muñoz, on behalf of the Stopping Transmission Of intestinal Parasites (STOP) project consortium

**Affiliations:** 1 ISGlobal, Hospital Clínic—Universitat de Barcelona, Barcelona, Spain; 2 Centro de Investigação em Saúde de Manhiça (CISM), Maputo, Mozambique; 3 Instituto de Ganadería de Montaña (CSIC-Universidad de León), Grulleros, León, Spain; 4 Departamento de Sanidad Animal, Facultad de Veterinaria, Universidad de León, Campus de Vegazana, León, Spain; 5 Fundación Mundo Sano, Buenos Aires, Argentina; 6 Instituto de Investigaciones de Enfermedades Tropicales, Universidad Nacional de Salta, Sede regional Orán, Argentina; 7 Departamento de Ciencias Biomédicas, Universidad de León, León, Spain; 8 Department of Medical Laboratory Science, College of Medicine and Health Science, Bahir Dar University, Bahir Dar, Ethiopia; 9 Departement of Parasitology, Leiden University Medical Center, Leiden, the Netherlands; 10 Eastern and Southern Africa Centre of International Parasite Control, Kenya Medical Research Institute (KEMRI), Nairobi, Kenya; 11 Faculty of Infectious and Tropical Diseases, London School of Hygiene and Tropical Medical Medicine, London, United Kingdom; 12 Pwani University Biosciences Research Centre (PUBRec), Pwani University, Kilifi, Kenya; 13 Instituto Nacional de Saúde (INS), Ministério da Saúde, Maputo, Mozambique; University of Cambridge, UNITED KINGDOM

## Introduction

More than 1 billion people have been reported to be infected with at least one soil-transmitted helminth (STH) worldwide, according to the last published report of the World Health Organization (WHO) [1]. WHO guidelines for STH control mainly encompass periodic administration of benzimidazoles (albendazole or mebendazole) to at-risk people of the endemic areas [1]. However, extended use of benzimidazoles could entail a great selection pressure for parasitic-resistant strains. In veterinary medicine, anthelmintic resistance in gastrointestinal nematodes has been developed in response to their excessive use, and it is currently considered a serious threat to livestock health and welfare [2, 3]. In humans, the estimated efficacy of albendazole and mebendazole against *Trichuris trichiura* has been observed to significantly decrease over time [4]. This observed decrement in drug efficacy could be due to the development of anthelmintic resistance (among other reasons such as drug quality and administration, the increasing of drug-efficacy studies, improvements in sensitivity of diagnostic tools after treatment, etc) after years of mass drug-administration campaigns, which is one of the major concerns in STH control [5].

Monitoring anthelmintic efficacy trials have been traditionally done by microscopic approaches, although it is well known that microscopy’s sensitivity may be insufficient in this context [6, 7]. We think that DNA-detection–based tools represent an accurate alternative to parasitological methods, and they should be evaluated and validated not only for monitoring worm burden before and after treatment but also for detecting genetic markers related to anthelmintic resistance.

## Monitoring infection intensity pre- and posttreatment

The cure rate (CR) and egg reduction rate (ERR) are both based on the microscopic detection of helminth eggs or larvae in stool samples and are traditionally used as indicators for monitoring drug efficacy. CR is calculated as the percentage of baseline-infected individuals that are diagnosed as negative posttreatment. ERR refers to the reduction in the number of eggs excreted posttreatment and is calculated using quantitative methods [6]. However, sensitivity, being microscopy’s major limitation, especially in posttreatment-occurring low-intensity infections, may overestimate both CR and ERR. Besides, they are observer dependent, which leads to variations in egg counts and limits standardisation options [7].

Recently, quantitative detection of parasite-specific DNA in clinical samples by real-time polymerase chain reaction (qPCR) tests has demonstrated substantial improvement in diagnostic performance as compared to microscopy, including the capability to differentiate between hookworm species [8, 9]. However, despite its high sensitivity, qPCR has not been frequently used for monitoring STH-infection intensity in drug-efficacy trials. Mejia and colleagues used multiparallel qPCR to assess *Ascaris lumbricoides* burden pre- and postalbendazole administration in a cohort of 125 children, wherein all the participants detected positive by qPCR at baseline were below the threshold of detection after 21 days of treatment [10]. Of late, efficacy of a single dose of albendazole against *A*. *lumbricoides* and *Necator americanus* was assessed with a pentaplex qPCR in context of a controlled deworming trial [11]. However, in this study, the egg number in stool samples was not determined; as a result, the authors were not able to correlate ERR with the infection intensity reduction rate based on qPCR results [11].

Highly sensitive diagnostic methods might allow the detection of light infections posttreatment and consequently aid the identification of potential resistant strains, particularly with respect to drug-efficacy trials. However, more systematic studies are required to further explore the exact relationship between observed parasite intensity determined by faecal egg counts and the quantitative outcome of the qPCR [12].

## Genetic markers associated with benzimidazole resistance

**Fig 1:**
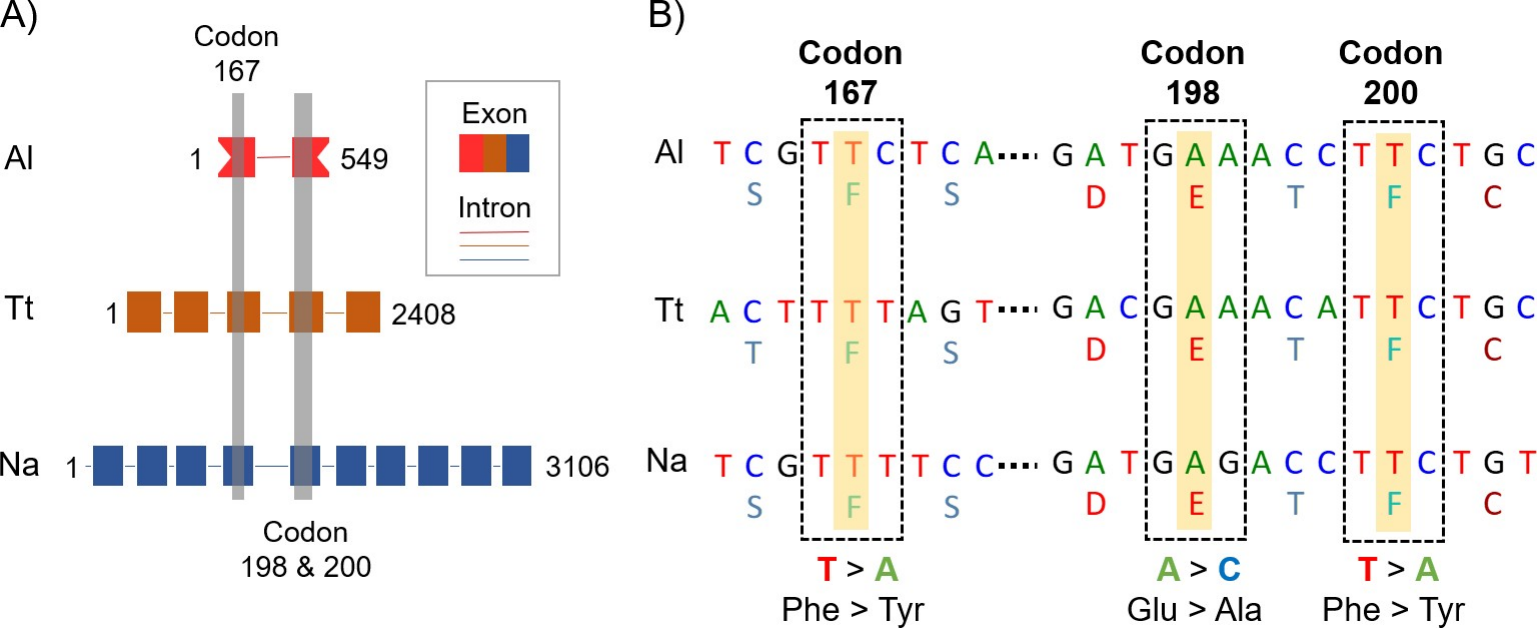
Putative benzimidazole-resistance SNP in human STHs. (A) Sequence information displayed in GenBank’s flat file; the boxes represent exons, whereas the lines represent introns. (B) Detailed nucleotide sequence of the three putative resistance SNPs and the amino-acid transcript at codons 167, 198, and 200. Al, *Ascaris lumbricoides* β-tubulin 1 sequence, partial cds (GenBank: FJ501301); cds, coding sequence; Na, *Necator americanus* β-tubulin 1 sequence, complete cds (GenBank: EF392851); STH, soil-transmitted helminth; Tt, *Trichuris truchiura* β-tubulin 1 sequence, complete cds (GenBank: AF034219).

As discussed above, qPCR might aid in evaluating drug efficacy by accurately estimating infection intensity before and after treatment [10–11]; however, the detection of genetic resistance markers could also predict treatment failures. Benzimidazoles bind to the colchicine binding site of β-tubulin, thereby disrupting microtubule polymerization and leading to parasite death. Parasite microtubules have been reported to be involved in vital functions at cellular level, including mitosis, motility, and transport [13]. In veterinary medicine, benzimidazole resistance is mainly associated with a single nucleotide polymorphism (SNP) at codon 200 of isotype 1 of β-tubulin–encoding gene, which results in amino-acid substitution of phenylalanine to tyrosine (F200Y). A similar SNP at codon 167 (F167Y) or a glutamate to alanine change at codon 198 (E198A) has been associated as a drug-resistance marker [3] (Fig 1). However, few studies have investigated the presence, frequency, and association of these markers with benzimidazole resistance in human STH species.

The presence of putative benzimidazole-resistance SNPs has been studied by different molecular methods [14–21], which are presented in Table 1.

**Table 1:** Characteristics of the molecular tools used for detecting putative benzimidazole-resistance SNPs in human STHs.

	Cost	Quantitative	Strengths	Weaknesses
**qPCR**	++	Yes	• Real-time detection • Widely used technique	• Expensive equipment
**RFLP–PCR**	+	No	• Simplicity • Widely used technique	• SNP detection limited by commercial endonucleases • Time-consuming procedure • Less accurate than the others
**SmartAmp**	+	No	• Isothermal amplification (no thermocycler) • Real-time detection • High amplification efficiency • Rapid and simple	• Need further validation
**Pyrosequencing**	+++	Yes	• High throughput • Multiple SNP detection • Accurate SNP frequency	• Highly expensive equipment • Not widely available

qPCR, real-time polymerase chain reaction; RFLP–PCR, restriction fragment length polymorphism-polymerase chain reaction; STH, soil-transmitted helminth

As observed, several molecular tools designed for detecting benzimidazole-resistant SNPs exist; however, technical and economic requirements could limit their application in low-income countries. SmartAmp technology has reported to be less expensive and highly efficient, and it has been also proposed as a quantitative technique [22] with potential applicability for mass screening in limited-resource settings. However, SmartAmp is still being validated, and currently only two studies describe this methodology for SNPs detection in STH (Table 2). The qualitative techniques that detect only the presence or absence of these SNPs possess a drawback: that the frequency of resistant alleles could not be measured in a pool of eggs. Conversely, pyrosequencing is more precise when compared with qualitative techniques, since it accurately determines multiple resistant allele frequencies in the same run. This technique has been widely used for detecting benzimidazole-resistant isolates in livestock nematodes [2], and therefore, we believe that pyrosequencing could be extremely helpful in understanding if there is a relationship between the presence of SNPs and their frequency with benzimidazole efficacy in STH, despite the high cost of pyrosequencing. It is expected that benzimidazole treatment could select resistant strains, and consequently, frequencies of resistant alleles would increase posttreatment.

**Table 2:** Summary of the results obtained by molecular tools in the detection of putative benzimidazole-resistance SNPs for human STHs.

	*A*.* lumbricoides*			*T*.* trichiura*			*N*.* americanus*			*A*.* duodenale*		
	F167Y	E198A	F200Y	F167Y	E198A	F200Y	F167Y	E198A	F200Y	F167Y	E198A	F200Y
**RFLP–PCR**	×	×	-	-	-	-	-	✓	✓	-	-	-	[18]
**qPCR**	-	-	-	-	-	-	✓	×	×/✓	-	×	×	[14, 21]
**SmartAmp**	✓	×	×	-	×	✓	×	✓	×	-	-	-	[19, 20]
**Pyrosequencing**	✓	×	×	✓	✓	✓	×	×	✓	-	-	-	[15–17]

F167Y, E198A, and F200Y: putative benzimidazole-resistance SNPs at codon 167, 198, and 200, respectively.

✓: method applied and SNP detected.

×: method applied and SNP not detected.

**-:** method not applied for SNP detection.

qPCR, real-time polymerase chain reaction; RFLP–PCR, restriction fragment length polymorphism-polymerase chain reaction; STH, soil-transmitted helminth

With regard to these techniques’ application, qPCR was first applied to detect the changes at codons 167 and 200 of *β*-*tubulin* gene of *N*. *americanus* [14, 21] and *Ancylostoma duodenale*, respectively [14]. More recently, restriction fragment length polymorphism (RFLP)-PCR has been used to study SNPs presence in *N*. *americanus* and *A*. *lumbricoides* populations in Brazil [18]. As an alternative, SmartAmp was developed for rapid and efficient genotyping of *β*-*tubulin* gene in *A*. *lumbricoides*, *N*. *americanus*, and *T*. *trichiura* [19–20]. Hence, pyrosequencing has been successfully used for determining the presence and frequency of three SNPs in all STH species except for *A*. *duodenale* [15–17]. Principal findings regarding genotypic benzimidazole resistance in STH are summarized in Table 2.

As previously described, all three SNPs (F167Y, E198A, and F200Y) have been detected in *T*. *trichiura* and *N*. *americanus*, whereas only F167Y has been observed in *A*. *lumbricoides*. However, most of these studies described only the presence or absence of SNPs and did not evaluate their association with the treatment response. However, so far only F200Y of *T*. *trichiura* has been noted to be associated with low response against albendazole [14, 15]. Diawara and colleagues measured the resistant allele frequency in *T*. *trichiura* by pyrosequencing in the stool samples collected from benzimidazole-naive populations [15] and albendazole-administered participants [16]. In nontreated areas, genotypic resistant profile (up to 63%) was noted, which explained the widespread suboptimal efficacy of benzimidazole against *T*. *trichiura* [15]. Moreover, a significant increase (from 3.1% to 55.3%) in F200Y frequency was observed posttreatment, which entailed the selection of resistant genotype [16]. The obtained results were in line with the findings observed in cases of veterinary helminths, wherein F200Y was reported as the most common SNP implicated in benzimidazole resistance [3]. However, this assertion must be cautiously interpreted, since the number of analyzed samples was low and the applied methodology was not harmonized across the study sites [16].

## The case of *Strongyloides stercoralis* and ivermectin

Despite being recognized as an important intestinal helminthiasis to incorporate into deworming campaigns, strongyloidiasis has been commonly neglected in STH interventions [21]. The inefficiency of microscopy to detect *S*. *stercoralis* and the latter’s unique feature of internal reproduction within the human host, makes worm burden less relevant, thereby rendering CR as a sole efficacy indicator [23]. Therefore, quantitative molecular methods such as qPCR would be even more important to accurately estimate the infection intensity pre- and postanthelmintic treatment.

Apart from this, albendazole displays low efficacy against *S*. *stercoralis*, and ivermectin is the drug of choice [24]. Ivermectin has been extensively used in mass drug-administration campaigns for onchocerciasis and lymphatic filariasis [25] and, together with albendazole, is being evaluated for STH deworming campaigns due to higher efficacy of the combination, particularly against *T*. *trichiura* [4]. Although, low response against ivermectin has been reported in veterinary helminths [2], we ignore the impact mass ivermectin administration (past and future) could entail in the development of anthelmintic resistance in *S*. *stercoralis* infections. Hence, in context of a potential widespread use of ivermectin against STH, we think that monitoring its efficacy and ivermectin-resistance emergence becomes of utmost importance, which has never been done so far.

## Conclusions

Early detection of resistant strains is crucial for the successful performance of drug-efficacy trials with anthelmintics. Despite a wide range of different molecular tools, not enough systematic studies have been reported that could definitively associate low drug efficacy by ERR with the presence of anthelmintic-resistance genetic markers.

The authors of this viewpoint joined efforts in 2018 for the development of the STOP project (Stopping Transmission Of intestinal Parasites), funded by the European & Developing Countries Clinical Trials Partnership (EDCTP), with the main aim of moving forward with the control and elimination of STH infections. To our knowledge, there are two similar projects that are evaluating molecular tools for monitoring drug-efficacy trials in human STH: the DeWorm3 project, which will test the feasibility of interrupting STH transmission using biannual mass drug administration targeting all age groups and with large scale application of PCR for monitoring drug-administration campaigns [26], and the Starworms study, with the overall aim of recommending the best diagnostic methods for monitoring drug efficacy and molecular markers to assess anthelmintic-resistance emergence in STH-control programs [22]. In contrast, STOP is built around a multicenter, randomized, clinical trial for evaluating safety and efficacy of fixed dose of ivermectin and albendazole coformulation in 1,800 STH-infected children in Mozambique, Kenya, and Ethiopia. During the development of the trial, we will evaluate DNA-detection–based assays for accurately estimating the efficacy of the new coformulation. Besides, we would determine the usefulness of this new drug combination against potential STH-resistant strains in comparison with albendazole. We believe that the study of genetic resistance should be incorporated in future evaluation of anthelmintics, especially in drug-efficacy trials, for a better understanding of the potential impact of this problem in the drug assessment.

## References

[1] World Health Organization. Soil-transmitted helminth infections. Available from: https://www.who.int/neglected_diseases/resources/who_wer9350/en/. [cited 2018 Oct 29].

[2] Martínez-Valladares M, Geurden T, Bartram DJ, Martínez-Pérez JM, Robles-Pérez D, Bohórquez A, et al Resistance of gastrointestinal nematodes to the most commonly used anthelmintics in sheep, cattle and horses in Spain. Vet Parasitol. 2015; 211 (3–4): 228–233. 10.1016/j.vetpar.2015.05.024 26112062

[3] Kotze AC, Hunt PW, Skuce P, von Samson-Himmelstjerna G, Martin RJ, Sager H, et al Recent advances in candidate-gene and whole-genome approaches to the discovery of anthelmintic resistance markers and the description of drug/receptor interactions. Int J Parasitol Drugs Drug Resist. 2014;4(3):164–84. 10.1016/j.ijpddr.2014.07.007 25516826 PMC4266812

[4] Moser W, Schindler C, Keiser J. Efficacy of recommended drugs against soil transmitted helminths: Systematic review and network meta-analysis. BMJ. 2017; 358, j4307 10.1136/bmj.j4307 28947636 PMC5611648

[5] Vercruysse J, Albonico M, Behnke JM, Kotze AC, Prichard RK, McCarthy JS, et al Is anthelmintic resistance a concern for the control of human soil-transmitted helminths? Int J Parasitol Drugs Drug Resist. 2011; 1 (1): 14–27. 10.1016/j.ijpddr.2011.09.002 24533260 PMC3913213

[6] World Health Organization. Assessing the efficacy of anthelmintic drugs against schistosomiasis and soil-transmitted helminthiases. 2013. Available from: http://apps.who.int/iris/bitstream/10665/79019/1/9789241564557_eng.pdf.[cited 2018 Oct 29].

[7] Vercruysse J, Behnke JM, Albonico M, Ame SM, Angebault C, Bethony JM, et al Assessment of the anthelmintic efficacy of albendazole in school children in seven countries where soil-transmitted helminths are endemic. PLoS Negl Trop Dis. 2011; 5, e948 10.1371/journal.pntd.0000948 21468309 PMC3066140

[8] Verweij JJ, Stensvold CR. Molecular testing for clinical diagnosis and epidemiological investigations of intestinal parasitic infections. Clin Microbiol Rev. 2014 4;27(2):371–418. 10.1128/CMR.00122-13 24696439 PMC3993103

[9] Easton AV, Oliveira RG, O'Connell EM, Kepha S, Mwandawiro CS, Njenga SM, et al Multi-parallel qPCR provides increased sensitivity and diagnostic breadth for gastrointestinal parasites of humans: field-based inferences on the impact of mass deworming. Parasit Vectors. 2016;9:38 10.1186/s13071-016-1314-y 26813411 PMC4729172

[10] Mejia R, Vicuna Y, Broncano N, Sandoval C, Vaca M, Chico M, et al A novel, multi-parallel, real-time polymerase chain reaction approach for eight gastrointestinal parasites provides improved diagnostic capabilities to resource-limited at-risk populations. Am J Trop Med Hyg. 2013;88:1041–7. 10.4269/ajtmh.12-0726 23509117 PMC3752800

[11] Vaz Nery S, Qi J, Llewellyn S, Clarke NE, Traub R, Gray DJ, et al Use of quantitative PCR to assess the efficacy of albendazole against *Necator americanus* and *Ascaris* spp. in Manufahi District, Timor-Leste. Parasit Vectors. 2018;11(1):373 10.1186/s13071-018-2838-0 29954461 PMC6025744

[12] Papaiakovou M, Gasser RB, Lttlewood T. Quantitative PCR-Based Diagnosis of Soil-Transmitted Helminth Infections: Faecal or Fickle? Trends Parasitol. 2019; 35(7): 491–500 10.1016/j.pt.2019.04.006 31126720

[13] Lacey E. The role of the cytoskeletal protein, tubulin, in the mode of action and mechanism of drug resistance to benzimidazoles. Int J Parasitol. 1988; 18: 885–936 10.1016/0020-7519(88)90175-0 3066771

[14] Schwenkenbecher JM, Albonico M, Bickle Q, Kaplan RM. Characterization of beta-tubulin genes in hookworms and investigation of resistance-associated mutations using real-time PCR. Mol Biochem Parasitol. 2007;156(2):167–74. 10.1016/j.molbiopara.2007.07.019 17850900

[15] Diawara A, Drake LJ, Suswillo RR, Kihara J, Bundy D, Scott ME et al Assays to detect beta-tubulin codon 200 polymorphism in Trichuris trichiura and Ascaris lumbricoides. *PLoS Negl Trop Dis*. 2009;3(3):e397 10.1371/journal.pntd.0000397 19308251 PMC2654341

[16] Diawara A, Halpenny CM, Churcher TS, Mwandawiro C, Kihara J, Kaplan RM, et al Association between response to albendazole treatment and β-tubulin genotype frequencies in soil-transmitted helminths. *PLoS Negl Trop Dis*. 2013;7(5):e2247 10.1371/journal.pntd.0002247 23738029 PMC3667785

[17] Diawara A, Schwenkenbecher JM, Kaplan RM, Prichard RK. Molecular and biological diagnostic tests for monitoring benzimidazole resistance in human soil-transmitted helminths. Am J Trop Med Hyg. 2013;88(6):1052–61. 10.4269/ajtmh.12-0484 23458960 PMC3752802

[18] Zuccherato LW, Furtado LF, Medeiros CDS, Pinheiro CDS, Rabelo ÉM. PCR-RFLP screening of polymorphisms associated with benzimidazole resistance in *Necator americanus* and *Ascaris lumbricoides* from different geographical regions in Brazil. PLoS Negl Trop Dis. 2018;12(9):e0006766 10.1371/journal.pntd.0006766 30222749 PMC6141064

[19] Rashwan N, Bourguinat C, Keller K, Gunawardena NK, de Silva N, Prichard R. Isothermal diagnostic assays for monitoring single nucleotide polymorphisms in *Necator americanus* associated with benzimidazole drug resistance. PLoS Negl Trop Dis. 2016;10(12):e0005113 10.1371/journal.pntd.0005113 27930648 PMC5145137

[20] Rashwan N, Scott M, Prichard R. Rapid genotyping of β-tubulin polymorphisms in *Trichuris trichiura* and *Ascaris lumbricoides*. PLoS Negl Trop Dis. 2017;11(1):e0005205 10.1371/journal.pntd.0005205 28081124 PMC5230752

[21] Orr AR, Quagraine JE, Suwondo P, George S, Harrison LM, Dornas FP. Genetic markers of benzimidazole resistance among human hookworms (*Necator americanus*) in Kintampo North Municipality, Ghana. Am J Trop Med Hyg. 2019;100(2):351–356. 10.4269/ajtmh.18-0727 30734697 PMC6367626

[22] Vlaminck J, Cools P, Albonico M, Ame S, Ayana M, Bethony J, et al Comprehensive evaluation of stool-based diagnostic methods and benzimidazole resistance markers to assess drug efficacy and detect the emergence of anthelmintic resistance: A Starworms study protocol. PLoS Negl Trop Dis. 2018;12(11):e0006912 10.1371/journal.pntd.0006912 30388108 PMC6235403

[23] Krolewiecki A, Nutman TB. Strongyloidiasis: A Neglected Tropical Disease. Infect Dis Clin North Am. 2019; 33(1):135–151. 10.1016/j.idc.2018.10.006 30712758 PMC6367705

[24] Krolewiecki AJ, Lammie P, Jacobson J, Gabrielli AF, Levecke B, Socias E, et al A public health response against *Strongyloides stercoralis*: time to look at soil-transmitted helminthiasis in full. PLoS Negl Trop Dis. 2013;7(5):e2165 10.1371/journal.pntd.0002165 23675541 PMC3649958

[25] Omura S, Crump A. Ivermectin: panacea for resource-poor communities? Trends Parasitol. 2014;30(9):445–55. 10.1016/j.pt.2014.07.005 25130507

[26] Ásbjörnsdóttir KH, Ajjampur SSR, Anderson RM, Bailey R, Gardiner I, Halliday KE, at al,; DeWorm3 Trials Team. Assessing the feasibility of interrupting the transmission of soil-transmitted helminths through mass drug administration: The DeWorm3 cluster randomized trial protocol. PLoS Negl Trop Dis. 2018; 12(1):e0006166 10.1371/journal.pntd.0006166 29346377 PMC5773085

